# Histological Investigation upon the Action of Arsenious Acids on the Nerve Fibres of the Pulp and the Dentinal Tissues

**Published:** 1883-09

**Authors:** C. F. W. Bodecker


### HISTOLOGICAL INVESTIGATIONS UPON THE ACTION OF ARSENIOUS ACIDS ON THE NERVE FIBRES OF THE PULP AND THE DENTINAL TISSUES.

BY DR. C. F. W. BODECKER.

During the session of the American Dental Association at Niagara, Dr. Bodecker laid before its members some of the results which he has obtained in his researches upon the action of arsenious acid on the tissues of the teeth. He said that the work had not been completed, but as the interest in this question was so great, he did not hesitate to communicate the results in part. This interest is evident from the protracted dispute concerning the action of the poison on the pulp alone, and the question whether it reaches further. Dr. Clows, he said, had been impressed by an unexplained phenomena; namely, that an application in the dentine showed action also in the pulp, an action similar to that induced by an application to the pulp directly, differing only in time. In 1869, Dr. Witzel, of Germany, studying the diseases of the tooth, observed a partial action on the pulp, the remainder being unaffected. In Germany this is largely accepted, whereas in this country it has been denied by many.

These and other instances from literature have influenced Dr. Bodecker in taking up the present line of investigation. But more especially was it his experience in sixteen cases in his own practice, which led him to inquire into the details of the process. Eleven of these cases he was enabled to follow up, and in all, the pulp stumps had died.

Dr. B. acknowledges that the human subject is the only final field of research, but from obvious reasons he has experimented also upon the teeth of animals. The drill holes were sunk into the dentine so that the poison was not in immediate proximity to the pulp. The teeth were examined after different periods of time, and the tissues were so treated by a preparation of osmic acid that changes from the action of reagents could be excluded.

The results were truly striking, and we shall mention them as briefly as possible, referring the reader for more details to a future time, when the investigations shall have been completed and published in full in this journal.

I. The pulp showed to the naked eye all symptoms of active inflammation.

II. Under the microscope the nerves appeared so changed that the myeline was broken up into small portions and granules.

III. When the action had been prolonged, there were found minute globules colored black by osmic acid, outside the nerve fibres, scattered in the surrounding tissue ; very probably a symptom of complete destruction of the medullated nerve fibers.

IV. In ground specimens (decalcification was in no case resorted to), the dentine was affected, starting either from the point of application, or radiating from different points of the pulp chamber. These places could be distinctly recognized by the naked eye as being intensely colored by the osmic acid.

V. The canaliculi were unequally enlarged, as were also their transverse off-shoots and connections. They were filled with black osmic-acid-colored masses, which appeared as if they were the broken up and enlarged contents of the canaliculi; (the Tomes fibers).

VI. The basis-substance is narrowed in those places where the canaliculi are enlarged. Certain peculiarities in coloring (picro-carmine) seem to indicate that the lime salts have been more or less extracted.

Dr. B. demonstrated these points at the blackboard, and showed the specimens under the microscope.

He called attention to the similarity of these changes to other diseases of the tooth, but distinctly stated that they must not therefore be thrown together, as the clinical aspect must also be brought into consideration.

He concluded by saying: "I am well aware that in very many cases the effect of the arsenic has apparently not been so bad as might be expected, but on the other hand you may all remember severe cases of local, or even constitutional disturbances, which could not be traced to any other cause than that of a dead tooth which had been treated with arsenious acid. Be all this as it may, the question is certainly in order whether, in view of the serious consequences, we are any longer justified in destroying pulps by the use of arsenious acid."

